# Intimate partner sexual and physical violence among women in Togo, West Africa: Prevalence, associated factors, and the specific role of HIV infection

**DOI:** 10.3402/gha.v7.23456

**Published:** 2014-05-26

**Authors:** Juan Burgos-Soto, Joanna Orne-Gliemann, Gaëlle Encrenaz, Akouda Patassi, Aurore Woronowski, Benjamin Kariyiare, Annette K. Lawson-Evi, Valériane Leroy, François Dabis, Didier K. Ekouevi, Renaud Becquet

**Affiliations:** 1 INSERM, Centre INSERM U897 ‘Epidémiologie et Biostatistique’, Bordeaux, France; 2 Université de Bordeaux, Institut de Santé Publique Epidémiologie Développement (ISPED), Bordeaux, France; 3 COMPTRASEC, CNRS UMR 5114, Pessac, France; 4 Service des maladies infectieuses, Centre Hospitalier Universitaire Sylvanus Olympio, Lomé, Togo; 5 Département de Santé Publique, Faculté mixte de médecine et pharmacie, Lomé, Togo; 6 Service de pédiatrie, Centre Hospitalier Universitaire Sylvanus Olympio, Lomé, Togo

**Keywords:** intimate partner violence, gender, HIV infection, Africa

## Abstract

**Background:**

A substantial proportion of newly diagnosed HIV infections in sub-Saharan Africa occur within serodiscordant cohabiting heterosexual couples. Intimate partner violence is a major concern for couple-oriented HIV preventive approaches. This study aimed at estimating the prevalence and associated factors of intimate partner physical and sexual violence among HIV-infected and -uninfected women in Togo. We also described the severity and consequences of this violence as well as care-seeking behaviors of women exposed to intimate partner violence.

**Methods:**

A cross-sectional survey was conducted between May and July 2011 within Sylvanus Olympio University Hospital in Lomé. HIV-infected women attending HIV care and uninfected women attending postnatal care and/or children immunization visits were interviewed. Intimate partner physical and sexual violence and controlling behaviors were assessed using an adapted version of the *WHO Multi-country study on Women’s Health and Life Events* questionnaire.

**Results:**

Overall, 150 HIV-uninfected and 304 HIV-infected women accepted to be interviewed. The prevalence rates of lifetime physical and sexual violence among HIV-infected women were significantly higher than among uninfected women (63.1 vs. 39.3%, p<0.01 and 69.7 vs. 35.3%, p<0.01, respectively). Forty-two percent of the women reported having ever had physical injuries as a consequence of intimate partner violence. Among injured women, only one-third had ever disclosed real causes of injuries to medical staff and none of them had been referred to local organizations to receive appropriate psychological support. Regardless of HIV status and after adjustment on potential confounders, the risk of intimate partner physical and sexual violence was strongly and significantly associated with male partner multi-partnership and early start of sexual life. Among uninfected women, physical violence was significantly associated with gender submissive attitudes.

**Discussion and conclusions:**

The prevalence rates of both lifetime physical and sexual violence were very high among HIV-uninfected women and even higher among HIV-infected women recruited in health facilities in this West African country. Screening for intimate partner violence should be systematic in health-care settings, and specifically within HIV care services. At a time of increased investments in couple-oriented HIV prevention interventions, further longitudinal research to better understanding of HIV-serodiscordant couple dynamics in terms of intimate partner violence is needed.

According to the World Health Organization (WHO), intimate partner violence is defined as the behavior, within an intimate relationship, that causes physical, sexual, or psychological harm or suffering (1). Several population-based surveys have reported that among all forms of violence against women, intimate partner violence is the most prevalent and it is considered the most common human rights violation hitherto (2, 3). Although there is still insufficient consensus on the operational definition and measurement of intimate partner violence, available estimations of lifetime prevalence of intimate partner violence vary from 15 to 71% worldwide, with the highest rates documented in resource-constrained settings (4). Intimate partner violence is associated with poor health outcomes among victims and is therefore a major public health issue (5–7).

Besides its dramatic physical and mental consequences, intimate partner violence has also been identified as an important risk factor for sexually transmitted diseases acquisition and particularly HIV infection (8). Intimate partner violence and HIV infection are linked by a very complex association, including diverse pathways. An increased risk of HIV transmission within abusive relationships and a greater likelihood of acquisition of HIV infection by violent husbands have been reported (8, 9). Acts derived from intimate partner violence, such as coerced sexual intercourse, during which women are unable to protect themselves from transmission, place women at direct risk of HIV infection (10).

Sub-Saharan Africa is the region reporting the highest epidemiological burden of HIV worldwide, with women accounting for 58% of newly acquired HIV infections (11). Furthermore, HIV infection in these settings spreads principally through heterosexual transmission and a substantial proportion of newly diagnosed infections occur in HIV-discordant cohabiting couples in which only one person is living with HIV (12). HIV prevention interventions aiming at protecting both couple members are thus a public health priority to reduce HIV incidence in these regions and several approaches have been already proposed (10–14). In spite of their proven efficacy, the challenges in implementing couple-oriented HIV preventive approaches are tremendous, and most importantly because they are based on mutual disclosure of HIV serological status (14–17). Intimate partner violence as one important consequence of HIV serological disclosure is thus one major limit of couple-oriented HIV preventive approaches and understanding its magnitude, dynamics, and factors associated are a prerequisite to the roll out of these couple strategies (1, 10, 12, 13).

In Togo, a West African country with an estimated prevalence rate of HIV infection of 3.4% nationwide and a predominantly heterosexual epidemic (11), intimate partner violence among women and its association with HIV infection has never been studied so far using a quantitative methodological approach, to the best of our knowledge. Moreover, data on intimate partner violence according to serological status in the West African region are very scarce, although it is needed to improve the scaling-up of HIV prevention, care, and treatment programs. This study aimed at estimating the prevalence and associated factors of intimate partner physical and sexual violence among HIV-infected and -uninfected women of childbearing age attending a clinical facility in Togo. We also described the severity and consequences of this violence as well as care-seeking behaviors of women exposed to intimate partner violence.

## Methods

### Study design and population

This project was an international collaboration between the INSERM (National Institute of Health and Medical Research) Research Centre U897 based in Bordeaux (France), the Sylvanus Olympio University Hospital in Lomé, and the non-governmental organization *Espoir Vie Togo*, one leading organization providing care and psychological support to people living with HIV/AIDS in Togo.

A cross-sectional survey was conducted between May and July 2011 within the Lomé University Hospital. The study population was constituted by volunteer participants attending clinical services in the hospital. HIV-infected women attending regular HIV care visits constituted the first group. Women attending postnatal care and/or children immunization visits, and who had been diagnosed HIV-uninfected during their last pregnancy, constituted the second group. For these two study groups, women aged at least 18 years, declaring having a current intimate partner or having ever had one, were eligible to recruitment.

### Study procedures

The survey was conducted in three stages. The first preparatory phase consisted of a methodological workshop between Togolese and French researchers to discuss the study design, validate the survey instruments, and address relevant ethical issues. A local team of four psychologists was recruited to adapt concepts described in the questionnaires to the local context, train interviewers on the study tool, ethical and privacy terms, and supervise data collection.

Second, a pilot study was carried out to test the acceptability, adequacy, and understandability of the recruitment procedures and questionnaire. Survey feasibility data and interviewer’s observations were integrated within the final survey procedures and tools.

Finally, every working day during the study period, each eligible woman was systematically asked to participate in the survey and to provide signed informed consent to be sequentially enrolled. Trained female health-care staff administered the questionnaire during a 20-min face-to-face interview in a private room.

All interviews were carried out in French, and all participants were provided information about local support services dedicated to women experiencing intimate partner violence. HIV-infected women were informed about the psychological support available at *Espoir vie Togo*.

The study protocol was approved by the National Ethic Committee of the Ministry of Health of Togo (N°0125/2011/MS/CAB/DGS/DPLET/CBRS). Written informed consent was obtained from all participants; and all data collection tools were strictly anonymous.

### Intimate partner physical and sexual violence assessment

Our questionnaire assessing intimate partner physical and sexual violence and controlling behaviors was largely inspired by the *WHO Multi-Country Study on Women’s Health and Life Events* questionnaire (version 10) (4, 18, 19).

All women participating in the survey were asked about their life experience on specific acts of physical and sexual violence induced by their intimate partner. Physical violence was defined as the use of physical force causing bodily harm (1). Within the survey, women were also asked about physical consequences of physical violent acts induced by an intimate partner, as well as help-seeking behaviors. Sexual violence was defined as any situation where women faced forced or coerced sexual act or attitude (1). Prevalence of physical/sexual violence was estimated as the proportion of women declaring having been exposed to any kind of physical or sexual violent act by their current intimate partner or any previous partner.

Additionally, we explored attitudes toward partner controlling behaviors that we summarized in six statements, illustrating different gender submissive situations to which women were asked to agree or not. We developed a scoring system to define a ‘submission index’ summarizing women’s attitudes toward partner controlling behaviors based on the sum of the number of positive answers to six questions detailed in Table 1.

**Table 1:** Characteristics of women interviewed according to their HIV status: Lomé, Togo, May–July 2011

	HIV uninfected (N=150)		HIV infected (N=304)		
		
	*n*	*%*	*N*	*%*	*p*
Women’s sociodemographic profile					
Age					
≥33 y/o	55	36.7	185	60.9	<0.01
<33 y/o	95	63.3	119	39.1	
Instruction level					
Not instructed	8	5.3	49	16.1	0.01
At least primary level	142	94.7	255	83.9	
Contraceptive method^a^					
Yes	97	64.7	128	42.1	<0.01
No	53	35.3	176	57.9	
Financial autonomy					
Employment					
Yes	103	68.7	242	79.6	0.01
No	47	31.3	62	20.4	
Financial autonomy to support herself^b^					
Yes	59	39.3	170	56.0	<0.01
No	91	60.7	134	44.0	
Modalities of entry to sexual life					
Age of first sexual intercourse					
≥18 y/o	63	42.0	113	37.2	0.32
<18 y/o	87	58.0	191	62.8	
Conditions of first sexual intercourse					
Consented	121	80.7	209	69.4	0.01
Coerced	29	19.3	92	30.6	
Women’s mental health					
Loss of interest^c^					
Yes	103	68.7	225	74.0	0.23
No	47	31.3	79	26.0	
Suicidal thoughts^d^					
Yes	36	24.0	157	51.6	<0.01
No	114	76.0	147	48.4	
Suicidal attempts^d^					
Yes	8	5.3	23	7.6	0.37
No	142	94.7	281	92.4	
Partners profile					
Polygamous					
Yes	19	12.7	138	45.4	<0.01
No	131	87.3	166	54.6	
Concurrent relationships					
Yes	52	34.7	203	66.8	<0.01
No	98	65.3	101	33.2	
Alcohol consumption					
Never/occasionally	85	56.7	125	41.0	0.01
Frequently	65	43.3	179	59.0	
Frequently involved in fights/riots					
Yes	11	7.3	65	21.4	<0.01
No	139	92.7	239	78.6	
History of non-partner violence					
Physical violence after 15 y/o					
Yes	60	40.0	200	65.8	<0.01
No	90	60.0	104	34.2	
Sexual violence after 15 y/o					
Yes	1	0.7	11	3.6	0.06
No	149	99.3	293	96.4	
Sexual violence before 15 y/o					
Yes	6	4.0	41	13.5	0.01
No	144	96.0	263	86.5	
Controlling behaviors and submission index					
A good wife obeys her partner, even if she does not agree with him					
Yes	113	75.3	278	91.5	<0.01
No	37	24.67	26	8.55	
It is important that a man shows his wife who is the boss?					
Yes	104	69.3	240	78.9	0.02
No	46	30.7	64	21.0	
A woman may have the freedom to choose her friends, even if her partner does not agree?					
Yes	19	12.7	49	16.1	0.33
No	131	87.3	255	83.8	
Satisfying her husband’s sexual desire even if she does not want to is a women’s duty?					
Yes	46	30.7	151	49.7	<0.01
No	104	69.3	153	50.3	
If a man abuses his wife, people around must intervene?					
Yes	122	81.3	240	78.8	
No	28	18.6	64	21.1	
A man must strike his wife if he considers this necessary?					
Yes	49	32.7	174	57.2	<0.01
No	101	67.3	130	42.7	
Submission index					
Median	3		4		<0.01

a Use of any contraceptive method at the moment of the survey.

b Having a financial autonomy to support herself and household without her partner for at least 1 month.

c During at least two weeks over the last 12 months.

d At least once over the last 12 months.

We also documented women’s sociodemographic characteristics (age, instruction level, and contraceptive use), data on the women’s partner (alcohol consumption and frequency of involvement in fights) and couple relationships (polygamy and concurrent relationship), women’s employment and financial autonomy (having a financial autonomy to support herself and household without her partner for at least 1 month), and the modalities of women’s entry in sexual life (conditions and age at first sexual intercourse). We also investigated women’s mental health (loss of interest, suicidal thoughts, and suicidal attempts) and the history of non-partner violence (physical violence after 15 years and sexual violence before and after 15 years).

### Statistical analysis

Sociodemographical and psychosocial characteristics of women and partners were described and compared between HIV-infected and -uninfected women. Maternal age was dichotomized into two categories with a cut-off defined by the median in the overall population (e.g. 33 years). Age of first sexual intercourse was dichotomized into two categories with a cut-off defined by the age at sexual majority in Togo (e.g. 18 years). The prevalence of lifetime physical and sexual violence was estimated separately among HIV-infected and -uninfected women and also compared between the two groups. Chi-square tests were performed to determine statistically significant differences. Sociodemographic and behavioral factors associated with lifetime physical and sexual violence in both groups were then assessed using logistic regression. Both univariate and multivariate analyses were carried out. Variables found to be statistically associated with intimate partner violence with a *p*-value of <0.25 were included in the multivariable model. To select the final adjusted model presented in this paper, we used a backward elimination method using a *p*-value of 0.05 (20). Adjusted odds ratios (aORs) were estimated using multiple logistic regression modeling and statistical significance was considered at the 5% level. Statistical analyses were generated using SAS software (version 9.2 for Windows, Copyright 2013 for SAS Institute Inc., Cary, NC, USA).

## Results

Overall, 454 women attending the Sylvanus Olympio University Hospital were informed about the study and screened for eligibility; all of them accepted to be interviewed and were included in the study. One hundred and fifty women were HIV-negative and 304 were HIV-positive.

### Study population characteristics

Sociodemographical and behavioral characteristics of women in both groups are described in Table 1 and summarized as follows.

Concerning women’s sociodemographic profile and financial autonomy, HIV-infected women were significantly older than uninfected women (35 [32–37] and 30 [29–34] years old in median [interquartile range], respectively, *p*<0.01). The proportion of HIV-uninfected women having completed at least primary education was significantly higher than among HIV-positive women (94.7 vs. 83.9%, *p*=0.01). HIV-infected women were significantly more likely than uninfected women to report some degree of financial autonomy (56 vs. 39.3%, *p*<0.001). HIV-uninfected women were more likely to be using a contraceptive method at the time of the survey than HIV-infected women (64.7 vs. 42.1%, *p*≤0.001).

Concerning modalities of entry to sexual life, the proportion of women having entered sexual life before the age of 18 was higher among HIV-infected than HIV-negative women (62.8 vs. 58.0%, *p*=0.32). HIV-infected women were significantly more likely to start sexual life through a first forced sexual intercourse than uninfected ones (30.6 vs. 19.3%, *p*=0.01).

Concerning partner’s profile characteristics, HIV-infected women were more likely to live within polygamous households than uninfected women (45.4 vs. 12.7%, *p*<0.001) and to declare that their partner had concurrent relationships out of the household (66.8 vs. 34.7%, *p*<0.001). Reported rates of partner alcohol consumption (59.0 vs. 43.3%, *p*=0.01) and partner involvement in fights and riots (21.4 vs. 7.3%, *p*<0.001) were more frequent among HIV-infected women than uninfected women.

Concerning women’s mental health, suicidal thoughts during the past 12 months were significantly more frequent among HIV-infected women than among the uninfected ones (51.6 vs. 24.0%, *p*<0.001). The proportion of HIV-infected women reporting loss of interest (74.0 vs. 68.7%; *p*=0.23) and suicidal attempts (7.6 vs. 5.3%, *p*=0.37) during the past 12 months tended to be higher than among uninfected ones but differences were not statistically significant.

Finally, HIV-infected women were slightly more likely to agree to submissive statements than HIV-uninfected women (scoring of 4/6 and 3/6, respectively; *p*<0.001).

### Intimate partner violence: prevalence, severity, and care-seeking behaviors

As detailed in Table 2, the prevalence rate of lifetime physical violence among HIV-infected women was 63.1% (95% CI: 57.5–68.4), significantly higher than among uninfected women (39.3%; 95% CI: 31.1–46.8; *p*<0.01). Similarly, HIV-infected women reported a significantly higher prevalence rate of lifetime sexual violence compared to the uninfected ones (69.7%; 95% CI: 63.8–74.1 vs. 35.3%; 95% CI: 27.3–42.6; *p<*0.01). The lifetime prevalence rate of both types of violence combined (physical and sexual violence) was 51.6% (95% CI: 45.3–56.6) among HIV-infected women and significantly higher than among uninfected women (18.6%; 95% CI: 11.8–24.1; *p<*0.01; Table 2).

**Table 2:** Lifetime prevalence rates of intimate partner vionce) among women, according to their HIV status: Lomé, Togo, May–July 2011

	HIV uninfected, *N*=150			HIV infected, *N*=304		
			
	*n*	%	95% CI	*n*	%	95% CI
Any form of physical violence						
Yes	59	39.3	31.1–46.8	192	63.2	57.5–68.4
No	91	60.7	52.1–67.8	112	36.8	30.6–41.3
Any form of sexual violence						
Yes	53	35.3	27.3–42.6	212	69.7	63.8–74.1
No	97	64.7	56.3–71.6	92	30.3	24.8–35.1
Any form of physical and sexual violence combined						
Yes	28	18.7	11.8–24.1	157	51.6	45.3–56.6
No	122	81.3	74.7–87.2	147	48.4	42.3–53.6

HIV-infected women were more likely to report a history of physical violence after the age of 15 than HIV-uninfected women (65.8 vs. 40.0%; *p*<0.001) and the frequency of sexual violence during childhood (before 15 years old) was 13.5% among HIV-infected versus 4.0% among uninfected ones (*p*=0.01; Table 1).

Among women ever victims of intimate partner physical violence (*n*=251), 194 (77.2%) reported physical injuries as a consequence of this violence (Table 3). Most commonly reported injuries were scratches and bruises (80.9%), dislocation and sprains (62.9%), eardrums rupture and black eyes (54.6%), penetration injuries and deep cuts (28.9%), and gashes and bites (20.1), although less commonly reported were burns (5.7%), fractures (4.6%), and broken tooth (1.6%). There were no differences between the type of injuries reported by HIV-infected and -uninfected women (data not shown).

**Table 3:** Distribution of physical injuries and care-seeking cascade reported by women victims of physical violence: Lomé, Togo, May–July 2011

	N	%
Number of physically injured women among those victims of physical violence (n=251)	194	77.2
Types of physical injuries reported (n=194)		
Scratches, hematomas	157	80.9
Dislocation, sprains	122	62.9
Eardrums rupture, black eyes	106	54.6
Penetration injuries, deep cuts, gashes	56	28.9
Bites	39	20.1
Burns	11	5.7
Fractures, broken bones	9	4.6
Broken teeth	3	1.6
Hospitalizations	14	7.2
Number of injured women needing medical care after being injured (n=194)	160	82.4
Number of injured women having received medical care when injured (n=160)	93	58.1
Number of women that told medical personnel the real cause of injuries (n=93)	49	52.6
Number of women referred to a dedicated support group (n=49)	0	0.0

Among the 194 women reporting being injured by intimate partner violence, 160 (82.4%) reported needing medical care for their injuries. From those needing medical care, 93 (58.1%) received medical care and 14 (7.2%) were even hospitalized. Among injured women in care, 49 (52.6%) disclosed real causes of injuries to medical staff and none of them was referred to local organizations to receive appropriate psychological support (Table 3).

### Factors associated with physical violence

The only common factor associated with a history of intimate partner physical violence regardless of women’s serological status was having a partner maintaining concurrent relationships out of the household (HIV-uninfected: aOR: 2.5; 95% CI: 1.1–5.5; *p*=0.02, and HIV infected: aOR: 2.2; 95% CI: 1.3–3.6; *p*<0.001). Otherwise, the profile of HIV-infected and HIV-negative women reporting intimate partner physical violence was different (Table 4).

**Table 4:** Factors associated with intimate partner physical violence according to their HIV status: Lomé, Togo, May–July 2011

	HIV uninfected						HIV infected					
		
	OR	95% CI	*p*	aOR	95% CI	*p*	OR	95% CI	*p*	aOR	95% CI	*p*
Women’s sociodemographic profile												
Age												
>33 y/o	1			1			1					
<33 y/o	2.02	0.99–4.09	0.05	0.39	0.17–0.88	0.02	0.93	0.58–1.50	0.78			
Education												
Not instructed	1						1			1		
At least primary level	1.58	0.38–6.58	0.53				1.99	0.99–3.99	0.05	2.05	1.00–4.19	0.05
Contraceptive methods^a^												
Yes	1			1			1					
No	1.65	0.83–3.27	0.15	2.28	1.03–5.01	0.04	1.05	0.65–1.68	0.84			
Modalities of entry to sexual life												
Age of first sexual intercourse												
>18 y/o	1			1			1					
<18 y/o	0.45	0.22–0.89	0.02	0.48	0.21–1.03	0.06	0.77	0.47–1.24	0.28			
Conditions of first sexual intercourse												
Consented	1			1			1					
Coerced	2.67	1.16–6.10	0.02	2.65	1.06–6.58	0.04	1.74	1.02–2.95	0.04			
Mental health												
Loss of interest^b^												
No	1						1					
Yes	1.07	0.52–2.16	0.86				2.90	1.71–4.91	<0.0001			
Suicidal thoughts^c^												
No	1						1					
Yes	1.53	0.72–3.27	0.27				1.75	1.09–2.80	0.02			
Suicidal attempts^c^												
No	1						1			1		
Yes	2.72	0.62–11.82	0.18				4.22	1.22–14.54	0.02	4.53	1.29–15.91	0.02
Partners profile												
Polygamous												
No	1						1					
Yes	1.86	0.70–4.89	0.21				1.48	0.92–2.37	0.1			
Concurrent relationships												
No	1			1			1			1		
Yes	2.86	1.42–5.73	0	2.51	1.13–5.52	0.02	2.37	1.45–3.88	0	2.21	1.33–3.65	<0.001
Alcohol consumption												
Never/occasionally	1			1			1					
Frequently	2.08	1.06–4.05	0.03	1.74	0.81–3.71	0.15	1.68	1.04–2.69	0.03			
Involved in fights/riots												
No	1						1					
Yes	4.6	1.16–18.12	0.03				2.06	1.10–3.82	0.02			
History of non-partner violence												
Physical violence after 15 y/o												
No	1						1			1		
Yes	0.93	0.47–1.82	0.84				1.51	0.93–2.46	0.09	1.54	0.92–2.56	0.1
Sexual violence after 15 y/o^d^												
Yes							1					
No							1.58	0.41–6.07	0.51			
Sexual violence before 15 y/o												
No	1						1					
Yes	8.33	0.94–73.22	0.06				1.7	0.81–3.54	0.16			
Financial autonomy												
Employment												
No	1						1					
Yes	0.64	0.31–1.28	0.21				0.93	0.51–1.66	0.8			
Financial autonomy to support herself^e^												
No	1						1					
Yes	0.77	0.39–1.51	0.45				0.92	0.57–1.47	0.74			
Submission index												
	1.18	0.89–1.53	0.24	1.33	0.97–1.80	0.07	1.13	0.91–1.39	0.28			

a Use of any contraceptive method at the moment of the survey.

b During at least two weeks for the last 12 months.

c At least once during last 12 months.

d Not enough subjects for the analysis among uninfected women.

e Having a financial autonomy to support herself and household without her partner for at least 1 month.

Among women’s sociodemographic and sexual characteristics, age was associated with physical violence only for HIV-uninfected women (33 years old or below vs. older than 33 years: aOR: 0.4; 95% CI: 0.2–0.9; *p*=0.02), and education level only for HIV-infected women (at least primary level vs. never attended school: aOR: 2.0; 95% CI: 1.0–4.2; *p*=0.05). Uninfected women not using any contraceptive method at the time of the survey were more likely to be victims of intimate partner physical violence (aOR: 2.3; 95% CI: 1.0–5.0; *p*=0.04). Among uninfected women, the odds of intimate partner sexual violence were significantly higher among those reporting a first coerced sexual intercourse (aOR: 2.6; 95% CI: 1.1–6.6; *p=*0.04).

In terms of women’s mental health status, having ever attempted suicide was strongly associated with a history of intimate partner physical violence, for HIV-infected women only (aOR: 4.5; 95% CI: 1.3–15.9; *p=*0.02). In the univariate analysis, loss of interest during at least two weeks (OR: 2.9; 95% CI: 1.7–4.9; *p<*0.001) and having had suicidal thought at least once over the last 12 months (OR: 1.7; 95% CI: 1.1–2.8; *p*=0.02) were also associated with having experienced intimate physical violence among HIV-infected women.

### Factors associated with sexual violence

As detailed in Table 5, starting sexual life before the age of 18 appeared to be the only common factor associated with intimate partner sexual violence among both HIV-infected and -uninfected women (HIV-infected women: aOR: 2.3; 95% CI: 1.1–4.9; *p*=0.01, and uninfected women: aOR: 2.3; 95% CI: 1.2–4.4; *p*=0.03).

**Table 5:** Factors associated with intimate partner sexual violence according to their HIV status: Lomé, Togo, May–July 2011

	HIV-uninfected						HIV-infected					
		
	OR	95% CI	*p*	aOR	95% CI	*p*	OR	95% CI	*p*	aOR	95% CI	*p*
Women’s sociodemographic profile												
Age												
>33 y/o	1						1					
<33 y/o	0.93	0.46–1.86	0.84				0.68	0.41–1.11	0.13			
Education												
Not instructed	1						1					
At least primary level	1.1	0.25–4.81	0.9				1.41	0.69–2.86	0.34			
Contraceptive methods^a^												
Yes	1						1					
No	0.7	0.34–1.43	0.33				1.23	0.75–2.01	0.41			
Modalities of entry to sexual life												
Age of first sexual intercourse												
>18 y/o	1			1			1			1		
<18 y/o	2.84	1.37–5.89	0.01	2.31	1.08–4.92	0.03	1.78	1.08–2.93	0.02	2.28	1.19–4.37	0.01
Conditions of first sexual intercourse												
Consented	1			1			1					
Coerced	2.34	1.02–5.33	0.04	2.18	0.92–5.18	0.08	2.87	1.54–5.34	<0.001			
Mental health												
Loss of interest^b^												
No	1						1					
Yes	1.93	0.89–4.14	0.09				7.51	4.26–13.24	<0.001			
Suicidal thoughts^c^												
No	1						1			1		
Yes	1.67	0.77–3.57	0.19				2.21	1.33–3.64	0	1.98	1.16–3.38	0.01
Suicidal attempts^c^												
No	1						1					
Yes	1.9	0.45–7.91	0.38				3.09	0.89–10.66	0.07			
Partners profile												
Polygamous												
No	1						1					
Yes	1.08	0.39–2.92	0.88				1.54	0.93–2.53	0.09			
Concurrent relationships												
No	1			1			1					
Yes	2.63	1.30–5.30	0.01	2.38	1.14–4.94	0.02	2.67	1.60–4.44	<0.001			
Alcohol consumption												
Never/occasionally	1						1					
Frequently	0.7	0.35–1.38	0.31				1.58	0.96–2.59	0.07			
Involved in fights/riots												
No	1						1			1		
Yes	2.35	0.68–8.09	0.18				2.87	1.39–5.93	<0.001	2.6	1.23–5.51	0.01
History of non-partner violence												
Physical violence after 15 y/o												
No	1						1					
Yes	0.46	0.22–0.93	0.03				2.84	1.70–4.72	<0.001			
Sexual violence after 15 y/o^d^												
Yes							1					
No							4.51	0.56–35.71	0.15			
Sexual violence before 15 y/o												
No	1						1					
Yes	3.88	0.68–21.91	0.13				2.83	1.14–6.99	0.02			
Financial autonomy^e^												
Employment												
No	1						1					
Yes	0.64	0.31–1.29	0.21				1.35	0.74–2.43	0.32			
Financial autonomy to support herself^a^												
No	1						1					
Yes	0.68	0.32–1.39	0.29				0.7	0.42–1.15	0.16			
Submission index												
	1.05	0.80–1.37	0.72				1.66	1.30–2.10	<0.001	1.58	1.23–2.03	<0.001

a Use of any contraceptive method at the moment of the survey.

b During at least two weeks for the last 12 months.

c At least once during last 12 months.

d Not enough subjects for the analysis among uninfected women.

e Having a financial autonomy to support herself and household without her partner for at least 1 month.

Otherwise, the profile of women reporting intimate partner sexual violence differed according to HIV serological status. HIV-uninfected women reporting having partners maintaining concurrent relationships out of household were more likely to report intimate partner sexual violence (aOR: 2.4; 95% CI: 1.1–4.9; *p*=0.02). For HIV-infected women, intimate partner sexual violence was associated with reporting suicidal thoughts (aOR: 1.9; 95% CI: 1.2–3.4; *p*=0.01), having partners who were involved in fights and/or riots with other men (aOR: 2.6; 95% CI: 1.2–5.5; *p*=0.01), and reporting a higher submission index (aOR: 1.6; 95% CI: 1.2–2.0; *p*<0.001).

## Discussion

We reported here on the prevalence of lifetime intimate partner physical and sexual violence according to HIV status among women recruited in health facilities in Togo. Our main finding is that the prevalence rates of both lifetime physical and sexual violence in 2011 were very high among HIV-uninfected women and even higher among HIV-infected women in this West African country. The prevalence rates we documented among uninfected women are similar to those estimated using the same methodology within population-based surveys from East Africa, both in terms of physical violence (32–49%) and sexual violence (23–58%) (4). On the contrary, more than half (63.1%) of the HIV-infected women in Togo reported lifetime intimate partner physical violence, which is almost twice as high than among uninfected women interviewed (39.3%) and much higher than the rates observed among HIV-infected women in Nigeria (6%) (21), or in eastern Africa (17%) (22). In terms of lifetime intimate partner sexual violence, the prevalence rate we documented among HIV-infected women in Togo (69.7%) is twice the rate of that among uninfected women and considerably higher than among other reported estimations in Africa, such as in Uganda (12%) (22). We reported here as well that more than half (51.6%) of the HIV-infected women had been victims of both types of violence, while this proportion was 18.6% among uninfected women.

Bruises were overall the most frequent injuries reported among African women victims of physical violence (23), but the proportion of Togolese women reporting serious and disabling injuries such as dislocations (62.9%) and deep cuts (28.9%) is alarming. The severity of the consequences of intimate partner violence is often underestimated. Indeed, in our study, a substantial proportion of women reported needing medical care after being severely injured, but only a few actually accessed medical care and, if they did, they rarely disclosed the real causes of their injuries. Finally, none of the injured women in our sample had been referred to existing organizations providing psychological support. These findings suggest that case detection of intimate partner violence should be systematically done by medical staff, and particularly within HIV care services. Clinical care evaluation checklists could include items related to physical and sexual violence to actively detect intimate partner violence. In addition, to ensure adequate case management, medical staff should be sensitized about intimate partner violence and consequences management and should be able to refer women to the appropriate supporting structures.

Although the prevalence of physical and sexual violence varied according to the HIV status among women of our sample, we identified three associated factors that are common to both groups. Reported partner multiple concurrent relationships were associated with higher rates of physical and sexual intimate partner violence regardless of serological status. Other African studies have reported similar findings, whereby women whose partner had several female partners were more likely to report sexual intimate partner violence and women suspecting their partner’s infidelity were at higher risk of any kind of violent act perpetrated by their male partner (12, 24–26). In Tanzania and South Africa, men acknowledging having multiple female partners confessed that being questioned about their fidelity could trigger physical and sexual violent acts against their female partner (27, 28). Gender norms in many African cultures expect masculine men to be in control of women, and this control can take the form of sexual multi-partnership and violent acts. On the other hand, the prevailing ideal of femininity in such contexts may prevent women from refusing these sociocultural patterns, and on the contrary seems to promote the acceptance of this behavior, increasing their risk of contracting HIV infection through sexual assaults (29). Multi-partnership, mostly among men, is an increasing HIV risk behavior in West African countries and the need to intensify behavior change efforts have been already pointed out (11). We believe that such efforts should focus on tackling masculinity construction, fostering women’s respect, and reducing gender inequality, all of which should be part of a comprehensive HIV behavior change preventive package.

We observed that starting sexual life before 18 years old was very frequent and appeared to be a risk factor of intimate partner violence for all women, regardless of their serological status. The high proportion of HIV-infected women experiencing a first forced sexual intercourse in our study is consistent with findings in South Africa (30). Since sexual abuse most often means unprotected sexual intercourse, these women may have been at the same time exposed to the risk of contracting HIV infection since very early ages and led into the vicious cycle of intimate partner violence (31). Age-appropriate sexuality education contributes to more responsible sexual behavior; nevertheless, gaps in basic knowledge about HIV and its transmission among young men and women remain important challenges (11). Sexuality education should be considered as a gateway to prevent HIV infection through a change of traditional gender norms based on fostering responsible and respectful sexual behaviors as early as possible in life.

Finally, prevailing submissive attitudes among Togolese women, expressed by an overall high acceptance of partner controlling behaviors and high submission index score, were associated with intimate partner violence, principally among HIV-infected women. Many women fearing intimate partner violence, even when aware of an HIV risk, may feel powerless to discuss infidelity, condom use, and HIV testing with their male partner (31). Renewed efforts are needed to foster women empowerment, including negotiation skills for safe-sexual practices addressed to women victims of intimate partner violence.

Several limits to our study need to be acknowledged. First, intimate partner violence may be a very sensitive subject for women and data collection was based on past experiences. We thus should not rule out a potential recall bias. To reduce information bias, however, interviewers were trained before conducting the survey and interviews were carried out in a private office. Further, due to the cross-sectional design of our study, we were not able to demonstrate a causal link between HIV infection and intimate partner violence or to explore the dynamic among these factors; however, our findings may have confirmed some of the factors to target when aiming at preventing intimate partner violence within clinical care services. Moreover, we did not present data on intimate partner psychological violence, as this would have required a thorough psychological assessment that could not be performed at the time of the survey. Finally, our study was conducted among a specific population of women attending a hospital facility in Lomé, and having been tested for HIV at least once in their life; thus, our results are not representative of all women in Togo.

One of the main strengths of our study, however, is its contribution to the pool of data available on intimate partner violence, data that can be compared to other settings as it was based on the use of *WHO Multi-Country Study on Women’s Health and Life Events* questionnaire. Our study highlights that intimate partner violence is a true public health issue in Togo, with a high social burden and severe health consequences on women, and especially among HIV-infected women. Our findings argue, in particular, for systematic case detecting of intimate partner violence – as well as any form of violence – within HIV services, to provide adequate medical care to women in need and to advise them about help-seeking strategies. Taking into account the high rate of HIV-discordant stable couples in sub-Saharan Africa, couple-oriented interventions are a priority among primary HIV preventive strategies (13), and because intimate partner violence, highly prevalent in West African contexts and one important consequence of HIV serological disclosure among HIV-infected women, is a major barrier to the acceptability of such approaches (12, 13), the assessment of intimate partner violence should be included in couple-oriented HIV preventive strategies.

Finally, tackling cultural representations and the social construction of masculinity and traditional gender norms in sub-Saharan African contexts is an important challenge to achieve behavior change in terms of sexual health and must be addressed from adolescent ages. Further longitudinal research is needed to understand HIV-serodiscordant couple dynamics with regard to intimate partner violence in African contexts and thus improve the acceptability and efficacy of couple-oriented HIV preventive interventions. Intimate partner violence, being highly prevalent in resource-constrained settings and a major public health concern, global health policies must turn more firmly to this issue.

## Statements:

The study protocol was approved by the National Ethic Committee of the Ministry of Health of Togo. All participants provided written informed consent.

This study was funded through the core grant of the INSERM U897 Research Centre in Bordeaux. Additional financial and logistical support was provided by the French National Agency on AIDS and viral hepatitis Research (ANRS, grant #12200). Juan Burgos-Soto was a PhD fellow of the French Ministry of Research. The sponsors had no role in study design and the collection, analysis, interpretation of data, the writing of the article, and the decision to submit it for publication.

The authors are indebted to the women who accepted to participate in this study. We wish to thank the staff of the Sylvanus Olympio Hospital for their assistance and particularly Rabiatou Darago who supervised the local team of psychologists to conduct the study.

This study was previously presented in part: Juan Burgos-Soto et al. Intimate partner sexual and physical violence according to HIV-status among women in Togo: prevalence and associated factors. 19th Conference on Retroviruses and Opportunistic Infections, Seattle, United States, March 2012 [Abstract # U-108].
